# Napabucasin Inhibits Proliferation and Migration of Glioblastoma Cells (U87) by Regulating JAK2/STAT3 Signaling Pathway

**DOI:** 10.3390/medicina60101715

**Published:** 2024-10-19

**Authors:** İlker Ünlü, İlhan Özdemir, Mehmet Cudi Tuncer

**Affiliations:** 1 Department of Neurosurgery, Faculty of Medicine, Beykent University, İstanbul 34398, Turkey; drilkerunlu@gmail.com; 2 Department of Gynecology and Obstetrics, Faculty of Medicine, Atatürk University, Erzurum 25240, Turkey; ilhanozdemir25@yandex.com; 3 Department of Anatomy, Faculty of Medicine, Dicle University, Diyarbakır 21280, Turkey

**Keywords:** glioblastoma doxorubicin, napabucasin, Stat3 inhibitory, MTT

## Abstract

*Background and Objectives*: Napabucasin (NP) was discovered as a natural compound that suppresses cancer stemness by inhibiting the signal transducer and activator of the transcription 3 (STAT3) signaling pathway. In this study, the anti-proliferative and apoptotic effects of NP and the chemotherapy agent doxorubicin (DX), a natural compound, on glioblastoma cells (U87) were investigated. *Materials and Methods*: In this study, the effects of NP and DX on cell viability on the glioblastoma U87 cell line were determined by MTT test. Expressions of Jak2/Stat3 genes were examined by qRT-PCR. Apoptosis was evaluated by Hoescht 33258 staining. Moreover, NP, its antagonistic–synergistic effects and IC50 doses of the combined treatment of DX were determined. *Results*: Napabucacin and doxorubicin were found to inhibit glioblastoma U87 cell proliferation. It was determined that NP applied in the range of 0.3–1 µM and its combination with DX killed almost all of the glioblastoma cells in 48 h of application. Additionally, it was observed that Jak2/Stat3 expressions downregulated. *Conclusions*: These results show that NP suppresses the proliferation of glioblastoma cells. It was shown that the combination of NP and DX can prevent invasion of the U87 cell line due to its Jak2/Stat3 inhibitory effect. Since it can suppress Jak2/Stat3, an important cancer cell proliferation pathway in glioblastoma, the combination of NP and DX can be used as an alternative treatment agent. But no synergistic effect of NP and DX on the U87 cells of the glioblastoma cell line was observed.

## 1. Introduction

One of the most common brain tumors is glioblastoma multiforme (GBM), a type of central nervous system cancer. GBM, which accounts for approximately 20–30% of primary brain tumors, is the most rapidly progressing and lethal tumor [1,2]. Early diagnosis of GBM and the prompt initiation of treatment are important for extending the patient’s survival. With simultaneous adjuvant radiotherapy and chemotherapy administered immediately after surgical resection following GBM diagnosis, the average survival time has been extended to a maximum of 18–24 months [3]. In previous studies, following combined therapy, the patient’s follow-up MRI scan revealed a recurrence of the tumor in the same location [4,5]. The difficulty encountered in the treatment of this malignant disease is due to both the complexity of the tumor itself and the numerous mechanisms it possesses against drug resistance.

The NP was discovered as a Stat3 inhibitor [6] and a natural naphthoquinone derivative. It is a molecule whose clinical studies are ongoing due to anticancer activities. It is a small molecule administered orally [7]. NP is an original molecule that inhibits Stat3, which controls important mechanisms such as the growth, proliferation and survival of cancer cells. Moreover, due to its ability to regulate the microenvironment, it suppresses the proliferation of cells representing cancer stemness. Recent studies report that this molecule acts as a drug that can bioactivate quinone oxidoreductase 1 (NQO1) [8,9]. Since it has been shown to have promising effects in animal xenograft studies [10,11], several preclinical studies have been conducted in patients with metastatic cancer [12,13]. BBI608 prevented the survival and self-renewal of cancer cells by targeting stem cells. Based on all these data, a multicenter phase I/II study showed that NP combined with pembrolizumab treatment in patients with metastatic colorectal cancer exhibits antitumor activity [14]. The number of studies highlighting the essential roles of Stat3 in neuroblastoma treatment is quite limited [15,16]. This active ingredient, a newly developed small-molecule Stat3 inhibitor, is an agent that can be administered orally. There are clinical studies of this inhibitor in combination with other chemotherapeutic agents [17,18].

Doxorubicin is an anthracycline-derivative antibiotic and an important antitumor drug with clinical application in the treatment of breast, ovary, testicular, thyroid and lung cancers and many sarcomas. Doxorubicin disrupts DNA replication and RNA transcription by intercalating in the DNA double strand and causes DNA damage by binding to the topoisomerase II enzyme. It is also a powerful antineoplastic drug used in the treatment of many types of cancer [19,20]. Although NP has been tested in phases as a good Stat3 inhibitor, studies of its side effects and the effects of its combinations with other chemotherapy drugs are very limited. It is important to develop alternative methods and conduct preclinical studies in order to reveal a more effective mechanism at lower doses. In this study, our aim is to explore the combined effects of NP and DX on the inhibition of proliferation in glioblastoma cells. Specifically, we seek to determine whether these agents exhibit synergistic or antagonistic interactions when used in combination. By elucidating the nature of these interactions, we aim to provide insights into optimizing therapeutic strategies for glioblastoma treatment.

## 2. Materials and Methods

### 2.1. Cell Culture

A U87 MG (ATCC, USA) glioblastoma cell line was used in the study. U87 MG cells were plated on 35 mm culture dishes 2 days before napabucasin and doxorubicin treatments. Cells were propagated in an incubator providing 37 °C temperature, 5% CO_2_ and 95% humidity conditions. Cells were used in the experiment when they reached 80–90% density. Confluent cells in 35 mm flasks were lifted with Trypsin-EDTA. A total of 3 × 10^5^ cells were seeded in 6-well plates in a DMEM medium containing 10% FBS, 2 mM L-glutamine, 100 U/mL penicillin and 100 μg/mL streptomycin. After a 5-day incubation, viable cells were counted using a hemocytometer for the combined treatment of NP (1 μM) and DX (0.7 μM).

### 2.2. MTT Assay

To determine cell viability, 5 × 10^3^ cells were seeded into a 96-well plate and these cells were allowed to grow for one day. Then, the cells were treated with different concentrations of DX and NP. For control, the cells were treated with DMSO (Merck, Rahway, NJ, USA). For this purpose, a 5 mg/mL dose of “Yellowtetrazolium MTT (3-(4, 5-dimethylthiazolyl-2)-2,5-diphenyltetrazolium bromide)” test solution was added into all wells at 20 µL/well. The DMSO was then added to the plates incubated with MTT solution for 4 h and incubated again for 4 h in the dark. Spectrophotometric reading was performed at 490 nm wavelength using a Multiskan GO microplate reader (ThermoScientific, Waltham, MA, USA) to determine the percentage of viability by detecting formazan formation. The wells to which the carrier was added were evaluated as the control group and the comparative viability ratio was performed according to these control wells.

### 2.3. Antagonistic–Synergistic Mechanism Detection

The stock solutions of NP and DX were prepared using ultrapure Ethanol (Merck, USA) and ultrapure water as 1:1. A total of 1mM stock solutions of prepared NP and DX were stored at −20 °C. The application was performed by reducing the final concentration of the carrier substance in the plate wells to 0.1%. In order to determine the antagonistic–synergistic effects and IC_50_ doses of NP and DX, U87 cells were seeded in 96-well culture dishes with multiple digital pipettes as 5 × 10^3^ cells. The cells that were left incubated overnight were allowed to adhere, and the next morning, single and combined drug applications were performed. In order to reveal the antagonistic–synergistic effect between NP and DX, serial dilution was performed starting from a 5 µM concentration, 8 different doses of the drugs were used in the application and 64 different doses were used with dosing squares. Plates were incubated for 48 h. After incubation, MTT test was performed for cell survival analysis. Each dose–response frame was performed in 96-well plate, and the experiment was repeated 5 times. The data obtained from the MTT analysis were used for Combenefit (Cambridge, UK) software to determine the antagonistic–synergistic effects of NP and DX and the resulting effects were determined. Each application was performed for 48 h and the appropriate doses were determined as a result.

### 2.4. Apoptosis

In order to detect apoptotic structures formed after apoptosis in the U87 cell line treated with NP and DX and to determine apoptosis, the nuclear stain Hoescht 33258 (ThermoScientific, Waltham, MA, USA), which stains according to DNA density and breaks, was used. For this, according to the staining procedure, 50,000 DNA per well was added to 24-well plates. Cells were seeded and incubated. After 48 h, NP IC50, DX IC50 and NP + DX IC50 agents were applied to these wells. Direct live cell staining was performed according to the kit protocol. After adding the staining agent, the cells were incubated at 37 °C for 30 min. The cells in the plates were then photographed using the Thermo EVOS^®^ FL ImagingSystem (Waltham, MA, USA) at ×20 magnification using brightfield mode and fluorescence mode.

### 2.5. Total RNA Isolation and cDNA Synthesis

The U87 cells were planted in 75 cm^2^ culture flasks and incubated until the logarithmic phase was reached. When the cells reached the logarithmic phase, vehicle control, DX IC_50_: 0.7 μM and NP IC_50_: 1μM doses were applied (DX + NP (0.7 + 1 µM)). RNA was isolated from the samples 48 h after the application. During the isolation phase, Purelink RNA mini kit (Thermo, Waltham, MA, USA) was used and the kit protocol was followed. The concentration was determined by Optizen NanoQ micro-volume spectrophotometer (Mecasys, Daejeon, Republic of Korea) and all doses were equalized with ultrapure water to 1000 ng/10 µL. Complementary DNA synthesis was performed to enable the RNAs obtained after the synchronization process to be amplified by PCR. At this stage, High-Capacity DNA Reverse Transcription Kit (Life Technologies, Carlsbad, CA, USA) was used. The obtained cDNAs were stored at −20 °C for ongoing studies.

### 2.6. Determination of Gene Expressions

In the study, Jak2 and Stat3 were divided into control and application groups of U87 glioblastoma cells. The expression levels of Jak2 and Stat3 genes were analyzed by qRT-PCR method. The following primers were used to detect the expression changes in the genes in 5′-3′ order (Table 1).

In gene expression studies, cDNAs obtained from RNAs isolated as described in the ‘Total RNA isolation’ section were used. These cDNAs were performed in qRT-PCR in accordance with the Pathway Scanner by Micromolecules qPCR Master Mix (Thermo, Waltham, MA, USA) protocol.

### 2.7. Protein–Protein Interaction (PPI) Analysis

PPI data were retrieved from the STRING database. The STRING database provides descriptions of protein–protein interactions (PPIs) and confidence intervals for data scores. A confidence score that was greater than or equal to 0.4 was chosen to construct the interaction network of proteins with target genes.

### 2.8. Statistical Analysis

The differences between relative gene expressions, live, dead and apoptotic cell ratios and averages in the control and treatment groups were determined using one-way ANOVA, and a significant difference was detected with the Tukey HSD test. In comparisons between the two groups, the independent sample T test was used, taking into account the homogeneity of the data. The results were found to be significant at *p* ≤ 0.05.

## 3. Results

### 3.1. Cell Viability (%)

**Figure 1:**
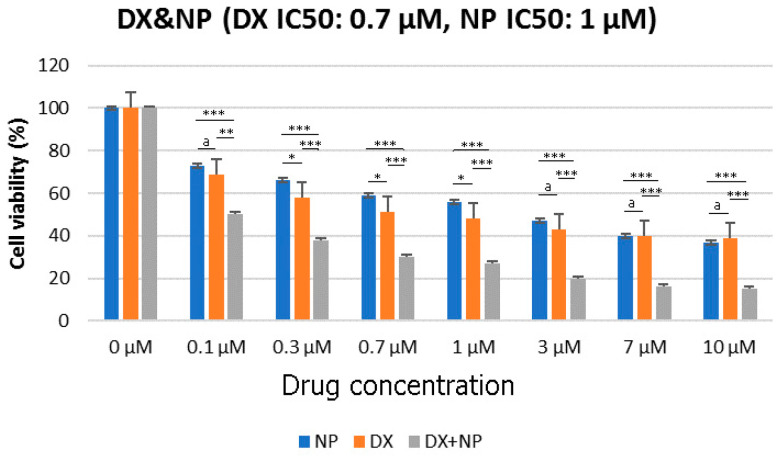
Effect of NP and DX at different concentrations on the survival rate of U87 cells for 48 h (a: *p* > 0.05, * *p* < 0.05, ** *p* < 0.001, *** *p* < 0.0001).

**Figure 2:**
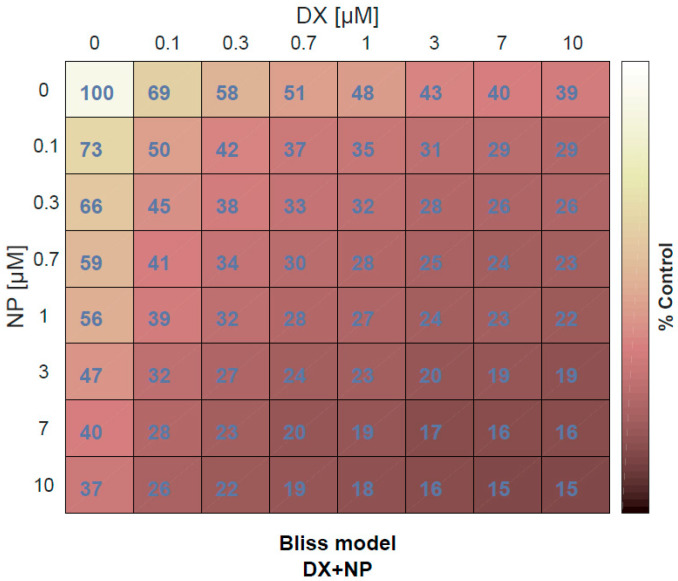
% Cell viability determined by MTT test of application of 64 different doses of DX and NP in U87 cell lines with dosing square starting from 10 µM concentration for 48 h.

The viability of U87 cells was evaluated by various assays after treatment with NP (1μM) or vehicle control (0.1% DMSO) for 48 h. As shown in Figure 1, napabucasin significantly reduced the proliferation of U87 cells in a dose-dependent manner. The most effective results in NP and DX applications were achieved within 48 h. For this reason, different dose results obtained up to 48 h are given in Figure 1. Additionally, the results revealed that DX had half-maximal inhibitory concentrations (IC_50_) of 0.7 μM and NP 1 μM in U87 cells (Figure 1). In the second step of the study, all eight different concentrations of DX and NP, prepared by serial dilution, were combined with each other and an x-response-square model was created by applying a total of 64 different doses (Figure 2). The MTT test was performed 48 h after the application and using a Combenefit software (2.02 version). The antagonistic and synergistic properties found on the death rates of glioblastoma cells were determined when these drugs were used together. The results are given in Figure 2.

### 3.2. Antagonistic–Synergistic Effect

**Figure 3:**
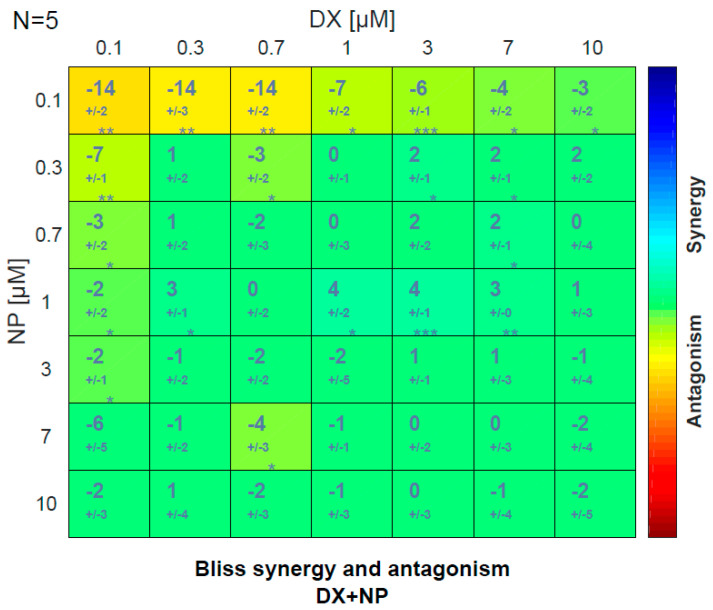
NP and DX for 48 h to determine the antagonistic and synergistic effect. (* *p* ≤ 0.05, ** *p* ≤ 0.01, *** *p* ≤ 0.001).

NP and DX applications were observed to determine a concentration-dependent killing effect on U87 cell lines. In combined applications, DX and NP used together significantly reduced the DX concentration required for the killing effect in glioblastoma cells. The same effects were seen significantly at all combined doses of DX + NP. When the two agents were applied alone or in combination, DX + NP did not show a synergistic effect according to the Bliss score at doses of (0.7 + 1) µM (Figure 3). This effect was also found to be statistically significant.

### 3.3. Apoptosis

**Figure 4:**
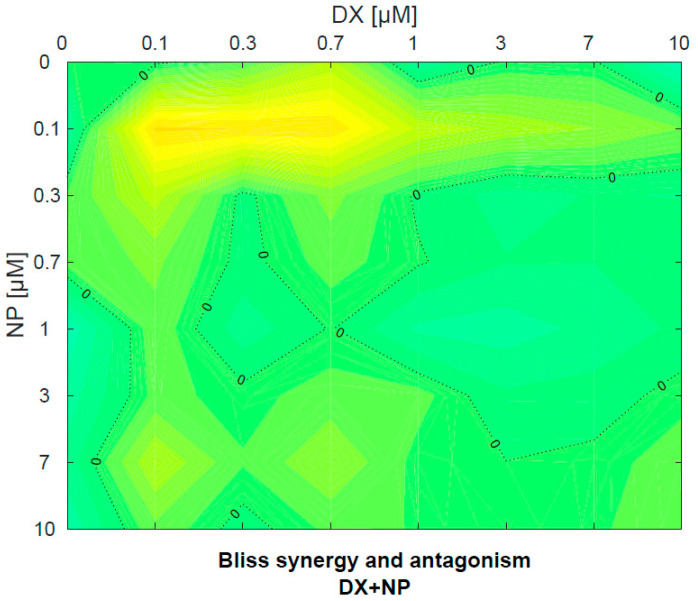
Contour view of the antagonistic or synergistic effect of NP and DX applied to U87 cell line.

**Figure 5:**
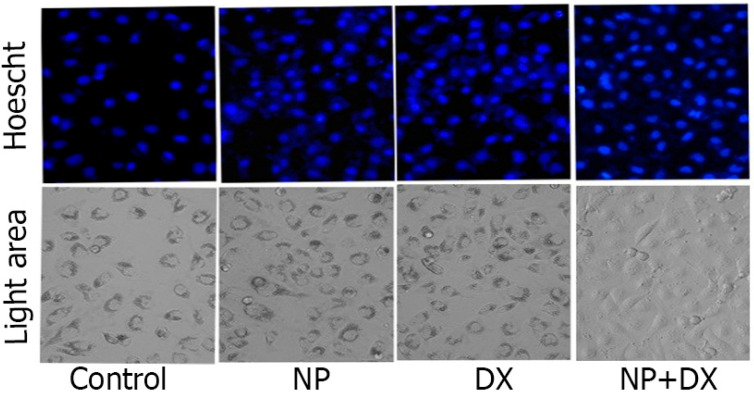
Vehicle control and U87 glioblastoma treated with NP IC_50_: 1 μM, DX IC_50_: 0.7 μM, NP + DX (1 + 0.7 µM) for 48 h. Cell morphology, nuclear structure and apoptotic body formation in cell populations (×20 magnification).

The apoptotic effects of NP and DX agents in the U87 cell line are shown in Figure 4 with nuclear morphological changes and apoptotic structures with Hoescht 33258-specific staining. DNA differentiation of the treatment agents in U87 cells was determined by apoptotic staining. While significant increases were detected in the number of apoptotic cells in the groups administered only NP and DX, the highest apoptotic activity was detected in the NP + DX combination (Figure 5).

### 3.4. Gene Expression

**Figure 6:**
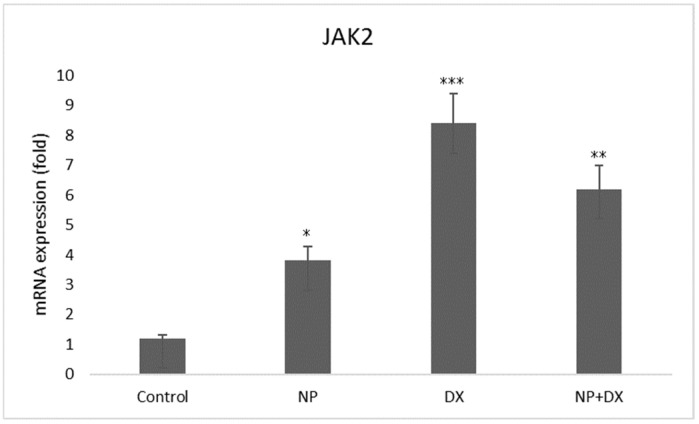
Relative fold increase values of Jak2 gene expressions 48 h after single and combined application of NP and DX in U87 cell line (data were normalized with β-actin and GAPDH mRNA levels by multiple control method, n = 6, data mean ± SE), * *p* ≤ 0.05, ** *p* ≤ 0.01, *** *p* ≤ 0.001.

**Figure 7:**
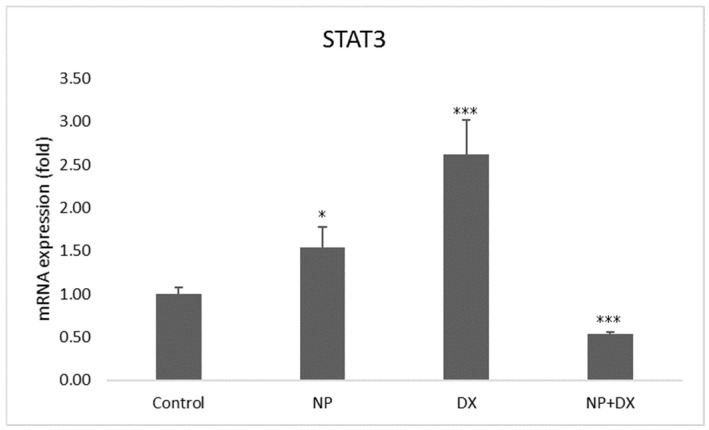
Relative fold increase values of STAT3 gene expressions 48 h after single and combined application of NP and DX in U87 cell line (data were normalized with β-actin and GAPDH mRNA levels by multiple control method, n = 6, data mean ± SE), * *p* ≤ 0.05, *** *p* ≤ 0.001.

Vehicle control, NP and DX agents were applied individually and in combination with glioblastoma cells; the U87 cell line and Jak2 and Stat3 gene expressions were detected after 48 h. The effects of NP and DX on Jak2 and Stat3 genes are shown in Figure 6 and Figure 7. According to these results, Jak2 increased with NP treatment, and statistical significance was determined when compared to the control group (*p* < 0.01). In contrast, the increase in Jak2 and Stat3 was determined to be statistically significant compared to the control group. In the DX group, the treatment’s Jak2 and Stat3 gene expressions increased significantly compared to the control (*p* < 0.001). While Jak2 upregulated significantly in the DX + NP group (*p* < 0.01), Stat3 downregulated significantly compared to the control group (*p* < 0.001) (Figure 6 and Figure 7).

### 3.5. PPI Analysis

**Figure 8:**
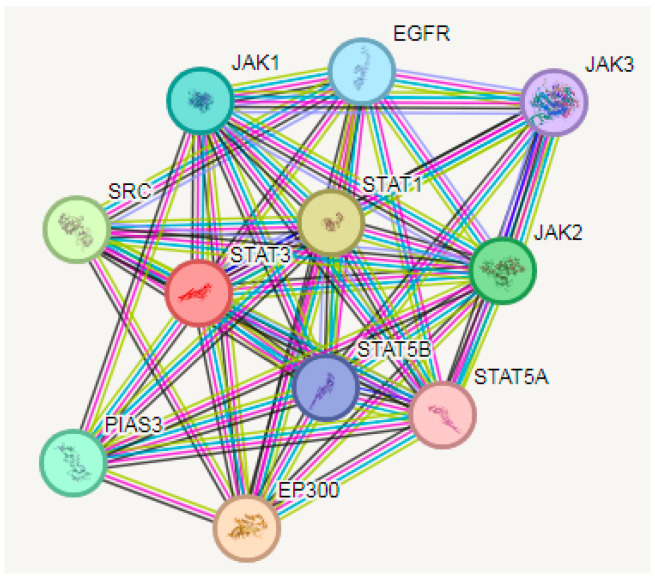
Interaction between various genes of glioblastoma.

Predictions from the STRING analysis were used to depict protein interactions. The visualization exhibits 11 nodes, 49 edges, and an average node degree of 8.91 (Figure 8). Based on the nodal degree, the following genes were identified as the top 10 central genes: EGFR, JAK1, SRC, PIAS3, EP300, STAT5A, STAT5B, JAK2, JAK3 and STAT1. These targets are hypothesized to be the primary targets in NP glioblastoma.

### 3.6. KEGG Enrichment Analysis

**Figure 9:**
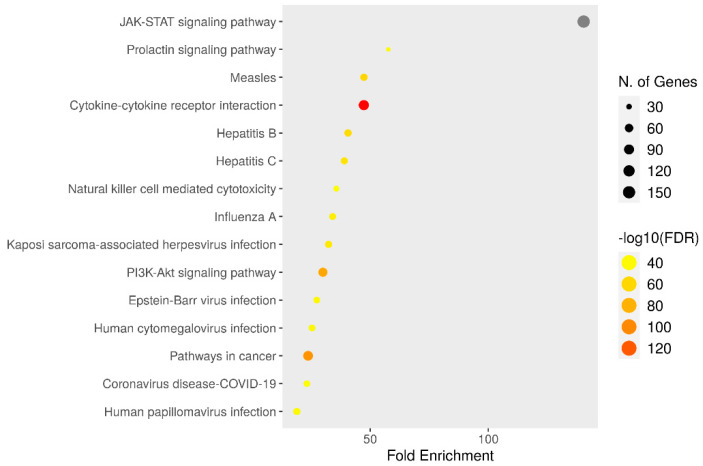
Enrichment analysis of the 150 common compound targets. Ninety of these targets are directly related to cancer signaling pathways.

KEGG enrichment analyses of target genes were performed with the Shiny 0.80 program. The findings showed that 150 genes were involved, and 90 pathways were cancer-related, exhibiting a significant correlation with target genes (*p* < 0.05) (Figure 9).

## 4. Discussion

Among brain tumors, glioblastoma multiforme (GBM), which is most commonly seen in adults, is among the most lethal tumors. Despite intensive research, the survival of GBM patients has only been extended to 24 months [21]. The most effective method used in GBM treatment is the surgical resection of the tumor followed by radiotherapy and chemotherapy, and 6 months after the tumor relapses in the area where it was removed [22,23]. It is important to develop new treatments to extend the life of these patients. The most important difficulty encountered in GBM treatment is that the patient does not respond to treatment due to resistance to temozolomide. Many studies have shown that these cells have heterogeneous structures as a result of examining cell lines obtained from biopsies taken from GBM tumors. Differences have been detected between the tumor-forming capacities of U118 and U87 cells obtained from GBM patients [24]. It has also been determined that these heterogeneous cells contain cancer cells with stem cell characteristics. These cancer stem cells cause the tumor to reoccur. Therefore, uncovering the mechanisms that prevent these cells from dying with aggressive combined treatment is an important step to target in GBM treatment. Currently, studies revealing the molecular mechanisms regulating glioblastoma pathogenesis are limited in number.

Although glioblastoma pathogenesis has been investigated for many years, the molecular mechanisms involved in this process have not yet been fully explained. Many studies have been conducted in the literature to reveal these mechanisms and these studies are still being intensively carried out. However, to date, a definitively effective treatment protocol for glioblastoma has not yet been fully established. Chan and colleagues, who analyzed miRNA expressions in glioblastoma cell lines, were the first researchers to investigate the functional properties of a single miRNA type. The researchers reported that miR-21 inhibition resulted in significantly increased apoptosis and, therefore, miR-21 may function as a micro-oncogene [25].

In an in vitro study, the anti-carcinogenic activity of NP was determined [26]. In addition to the primary antioxidant effects of these compounds, it was also determined that they had a broad spectrum of biological effects on carcinogenesis. It has been stated that the anti-proliferative activities—and therefore, potential anticancer effects of phenolic compounds—originate from their aromatic rings and hydroxylic groups [27]. In our study, it was determined that NP had an anti-proliferative effect on the glioblastoma cell line. There are very limited sources showing the use of NP in cancer studies. In this study, we found that NP reduced the proliferation of U87 cells and inhibited proliferation.

The newly discovered NP is reported as an effective Stat3 inhibitor. Li and colleagues reported that NP inhibits the Stat3 gene and prevents metastasis in many cancers. It was also reported that NP can effectively suppress. Subsequent studies reported that NP treatment disrupts the spherical formation of cancer stem cells, which are the cause of metastasis in liver cancer, and reduces stem cell markers such as SOX2, BMI-1, Nanog and c-Myc by inhibiting stem cell genes. The combination of NP with chemotherapy agents and traditional alternative compounds has reported remarkable results in preclinical data [28]. We confirmed the role of Stat3 in improving the poor progression and prognosis of glioblastoma. Treatment of U87 cell line with NP and DX significantly increased cell proliferation via the Jak2/Stat3 signaling pathway. We found that it was blocked. We also found that NP treatment significantly reduced the expression and phosphorylation of Stat3, an important transcription factor of the Jak2 signaling pathway.

There are animal model studies on the anticancer effects of NP. As a form of monotherapy, NP administration has been reported to inhibit and reduce tumor volume in xenograft mouse models of osteosarcoma, hepatocellular carcinoma, and human acute myeloid leukemia [27,29,30]. In another group of studies, it was reported that it extended the lifespan of mice in glioma and melanoma mouse models [29,31]. Additionally, napabucasin treatment has been reported to inhibit bone osteolysis and bone resorption in a 4-week-old female BABL/c nude mouse model, metastasis in an osteosarcoma model, and a pancreatic cancer xenograft model [27,28]. In line with these data, the main mechanisms of napabucasin’s in vivo anticancer effects include inhibition of cancer stem cells, inhibition of proliferation and induction of apoptosis.

In order to improve the quality of life of glioblastoma patients after chemotherapy and prolong their survival, it is necessary to prevent migration in tumor cells, overcome resistance to chemotherapeutic drugs, prevent recurrence and reduce side effects. This depends only on the development of more effective and, when necessary, combined treatment strategies [32]. There is no study in the literature investigating Stat3 expression after NP and DX treatment used in glioblastoma treatment. In this study, Jak2/Stat3 expressions were examined for the first time in the glioblastoma cell line (U87) after a separate and combined application of NP and DX. As a result of our study, the significantly decreased Stat3 level in the 48-h NP + DX combined treatment group compared to the other groups demonstrated the effectiveness of the combined treatment. In addition, STAT3 expression in glioblastoma should be proven by in vivo studies.

## 5. Conclusions

In this study, we showed that NP and DX combination have anti-proliferative and anti-migration effects on the U87 glioblastoma cell line. We also observed that there was no synergistic or antagonistic effect of the combined treatment of NP and DX on the U87 cell line. However, NP treatment suppressed the proliferation of U87 cells when applied together with DX. We observed that NP suppressed the levels of Jak2/Stat3, which regulates the growth and proliferation of cancer cells, but further studies are needed to determine the intracellular mechanisms through which it exerts this effect. Furthermore, in vivo studies are needed to refine the results.

## Figures and Tables

**Table 1:** Primers of genes analyzed by qRT-PCR method in 5′-3′ order.

**JAK 2: F: CAGTGGTCAAGAGGGAAACA, R: TGTCTGAGCGAACAGTTTCC**
**STAT3: F: GGAGGAGTTGCAGCAAAAAG, R: TGTGTTTGTGCCCAGAATGT**
**β-actin: F: CCTCTGAACCCTAAGGCCAAC, R: TGCCACAGGATTCCATACCC**

## Data Availability

The original contributions presented in the study are included in the article, further inquiries can be directed to the corresponding author.
